# The combined effects of temperature and exogenous bacterial sources on mortality in the Eastern oyster (*Crassostrea virginica*) under anoxia

**DOI:** 10.1007/s00227-025-04617-4

**Published:** 2025-03-17

**Authors:** Laura Steeves, Keryn Winterburn, Michael R. S. Coffin, Jose M. F. Babarro, Thomas Guyondet, Luc A. Comeau, Ramón Filgueira

**Affiliations:** 1 Flødevigen Research Station, Institute of Marine Research, Flødevigen, His Norway; 2 Biology Department, Dalhousie University, Halifax, NS Canada; 3 Fisheries and Oceans Canada, Gulf Fisheries Centre, Moncton, New Brunswick, Canada; 4 Instituto de Investigaciones Marinas, IIM-CSIC, Vigo, Spain; 5 Marine Affairs Program, Dalhousie University, Halifax, NS Canada

**Keywords:** LT_50_, Eastern Oyster, Bacteria, Temperature, Mortality, Low oxygen

## Abstract

In aquatic environments, low dissolved oxygen concentrations can result in depressed bivalve defense systems while promoting anaerobic bacterial growth, ultimately leading to increased bivalve mortality rates. Although the relationship between low oxygen availability and bivalve mortality has been previously examined, the mechanisms of mortality remain not well understood, limiting our ability to predict mass mortality events. In this study, the effect of anoxia (< 0.1 mgO_2_L^−1^) on adult oyster (*Crassostrea virginica*) mortality rates was explored experimentally using a factorial design, which included the effect of temperature (20°C vs. 28°C) combined with the presence/absence of an exogenous bacterial source (anoxic sediment vs. sterile sediment). Additionally, the effect on oyster mortality rate of removing vs. not removing deceased oysters from the experimental chambers was assessed. Oyster mortality rates, estimated as the time taken for half of the population to die (LT_50_) in anoxic conditions were significantly affected by temperature, the presence of anoxic sediment, and experimental execution (removing vs. not removing deceased oysters). Temperature had the greatest effect on mortality overall, with high temperatures resulting in increased mortality rates, whereas the presence of anoxic sediment only increased mortality rates consistently at high temperatures. The results of this study suggest that bacterial sources play a role in the mortality rate of oysters under warm anoxic conditions.

**Supplementary Information:**

The online version contains supplementary material available at 10.1007/s00227-025-04617-4.

## Introduction

Marine environments have been experiencing an overall decline in dissolved oxygen concentrations from the middle of the twentieth century when most monitoring efforts began (Keeling et al. 2010; Breitburg et al. 2018). Declining oxygen concentrations may be driven by increasing water temperatures; it has been estimated that as much as 15% of oxygen loss in the ocean results from the decreased oxygen solubility in warming water (Helm et al. 2011); relatedly, as temperatures rise, so too does biological oxygen demand (Pernet et al. 2007; Brown et al. 2016; Khan et al. 2018). In coastal waters, an increase in nutrients primarily from agricultural run-off sewage discharge may cause eutrophication, another process that has been directly linked to lowered oxygen concentrations (Justić et al. 1993; Breitburg et al. 2018). Eutrophication can contribute to low oxygen conditions through increases in algal and plant growth in surface waters, increasing the export of organic material to the seafloor and stimulating microbial oxygen consumption (Rabalais et al. 2014; Boyd 2020). Decreased oxygen availability in coastal waters may cause increased stress, disease, and ultimately mortality in ecologically and economically important species, including marine bivalves. The reduction of oxygen availability during aerobic respiration triggers a reduction in metabolic rate, and related transcription factors that minimize cellular stress during metabolic depression (Kurochkin et al. 2009). When aerobic metabolism cannot be maintained, bivalves may switch to anaerobic metabolism, leading to a further reduction in metabolic rate (de Zwaan and Wijsman 1976; Babarro and de Zwaan 2002; King et al. 2019). This reduction in metabolism may cause a decrease in hemocyte counts, affecting the ability of bivalves to eliminate foreign organisms (Paschke et al. 2010). This, in turn, may cause an increased susceptibility to bacterial infection in bivalves as a result of their compromised immune system (Babarro and de Zwaan 2002; Kurochkin et al. 2009).

Under severe hypoxia (< 0.5 mgO_2_ L^−1^), anaerobic bacteria are able to proliferate and infect bivalve’s tissues (Babarro and de Zwaan 2002). Increased mortality rates in bivalves may be observed when high bacterial loads coincide with immune system depression (Babarro and de Zwaan 2002; Paschke et al. 2010). This mortality is often an indirect consequence of low oxygen conditions, where associated bacteria cause tissue necrosis (de Zwaan and Babarro 2001; Babarro and de Zwaan 2002; Coffin et al. 2021). The effect of severe hypoxia and anoxia on bivalve survival has been widely studied with experiments typically conducted under sterile laboratory conditions in which exogenous bacterial sources not associated with the bivalve are removed. In addition to the lack of natural sediment in experimental chambers, water is usually sterilized in these experiments (e.g., Stickle et al. 1989; Anderson et al. 1998; de Zwaan and Babarro 2001; Babarro and de Zwaan 2002). However, both natural and farmed bivalves grow in water with exogenous bacterial sources (Kai et al. 2017). Another source of bacteria often not considered in the experimental design is that from surrounding deceased and decaying bivalves as they are often removed for tissue analysis (e.g., de Zwaan et al. 2001; Khan et al. 2018). However, previous experiments indicate that necrotic tissue may act as a substrate for bacterial proliferation resulting in increased bivalve mortality (de Zwaan and Babarro 2001). Additionally, in natural conditions, anoxia often coincides with high temperatures, which may result in increased virulence of bacteria due to the increased transcription of bacterial virulence genes, therefore increasing infection rate in bivalve hosts (Babarro and de Zwaan 2002; Guijarro et al. 2015; Khan et al. 2018).

As anoxic events are expected to occur more frequently in coastal waters due to climate change, it is important to understand how to manage these events for both wild and farmed species. However, previous laboratory studies examining the relationship between anoxia and mortality in bivalves have not fully captured the complexity of interacting environmental stressors that result in mortality (e.g., high temperature, bacteria). Accordingly, the objective of this research was to determine the effect of exogenous bacteria from natural sediment and the presence of dead oysters, which represent an additional substrate for bacterial proliferation, on oyster mortality rate at different temperatures under anoxia. In addition to measuring mortality rate, DNA characterization of the bacterial community in the oyster tissue was conducted to determine if bacterial communities differed in composition or relative abundance differences emerged between treatments. The results of this study will allow for a more accurate understanding of the factors determining oyster survival under anoxia.

## Materials and methods

### Oysters

Cultured adult *Crassostrea virginica* (3 years old, 60 ± 5 mm) were collected from Sober Island Pond in Nova Scotia (44° 50′ 33″ N, 62° 28′ 1″ W), on September 20th, 2018 (experiment-1) and October 18th, 2018 (experiment-2). All oysters were hatchery produced diploids. Oysters were transported on ice to Dalhousie University, a 2 h drive from Sober Island Pond, and held in the Aquatron facility in flow-through tanks of 50 µm sand-filtered and aerated seawater at 17 °C. All lights were off in the laboratory room unless daily maintenance was being performed (~ 1 h day^−1^). Water was pumped from 9–12 m depth and salinity was stable (~ 30 ppt) throughout the duration of the experiment. Prior to beginning the experiment, all oyster shells were scrubbed to remove organic matter and oysters were acclimated to either 20 °C or 28 °C. These temperatures were chosen as the average summer water temperature (20 °C), and the highest recorded water temperature in estuaries of the southern Gulf of St. Lawrence (28 °C) (Fisheries and Oceans Canada, 2018). The Gulf of St. Lawrence was chosen for reference temperatures as an area in this region, Tracadie Bay, New Brunswick, has *C. virginica* farms that have previously experienced anoxic events and mass mortalities (Department of Fisheries and Oceans Canada, 2018). Water temperature in the laboratory was increased from 17 °C to the experimental temperature at a rate of 2 °C day^−1^ to avoid physiological stress (Casas et al. 2018).

### Sterile and anoxic sediment

Sterile sediment (playground sand) was washed to remove soluble inorganic and organic compounds, dried, and placed in a furnace for 4 h at 450 °C to sterilize it and remove any remaining organic materials. The sediment was cooled in a sterile environment to room temperature prior to use in the experiments.

Anoxic sediment was collected from Tracadie Bay, New Brunswick (47° 32′ 45″ N, 64° 52′ 49″ W) and shipped overnight to Dalhousie University in a cooler with ice the day prior to commencing the experiments. This protocol was designed to minimize potential microbial community changes that may have happened during collection and transport. The sediment was retrieved directly from the top 5 cm layer of an oyster aquaculture site with a history of anoxic conditions. Both anoxic and sterile sediments (360 g chamber^−1^) were homogenized and distributed in individual experimental chambers to achieve approximately a 1-cm depth of sediment. Sediment was not disturbed during the experiments.

### Experimental design and oyster survival

Four independent water baths (21.5L) were used for both experiments, each containing four isolated experimental chambers (4.6L each, 16 chambers in total). A factorial design was used to study the effect of two factors: temperature (20 °C vs. 28 °C) and exogenous bacterial sources (sterile sand vs. anoxic sediment), where each combination of treatments was replicated in an experimental chamber in each water bath (4 independent replicates per treatment). Six oysters were placed in each experimental chamber (~ 0.5 g oyster tissue dry weight L^−1^), resulting in 24 oysters per treatment. Severe hypoxic water (< 0.1 mgO_2_L^−1^; henceforth anoxic) created in a common header tank by bubbling N_2_ through UV-sterilized seawater was used to fill the chambers. This low concentration of oxygen was selected to mimic field conditions from oyster farming areas in Atlantic Canada where mass mortality events may occur (Coffin et al. 2021). Once anoxia was achieved (as measured with an optical oxygen sensor PreSens Fibox 3 trace v3) the chambers were sealed and maintained at their respective temperatures (20 °C or 28 °C) throughout the experiment by circulating water through the water baths. No food was provided during the experiment. The chambers were static with no renewal of water during the experiment. The only exception to this was that during the removal of deceased oysters or water from the tanks (experiment-1, detailed below), additional anoxic sterile water at the respective temperature, was added to top-up the chambers to the same initial level to remove the air layer created due to volume change. In addition to the four water baths, a flow-through (sand-filtered normoxic water) tank with 6 oysters at 20 °C with no addition of any type of sediment or food was used to ensure that cleaning, handling, and transport stress did not contribute to oyster mortality. Given that no mortality was observed in the normoxic condition, it was assumed that mortality in the experiments was a result of the experimental treatments. At the end of the experiment, these oysters were also measured for whole dry tissue weight (0.35 ± 0.15 g mean ± SD) to observe the general condition of the oysters, and to make estimates of oyster biomass (in dry tissue weight) per experimental chamber.

Dissolved oxygen was monitored daily throughout the experiment using an optical oxygen sensor (PreSens Fibox 3 trace v3). The temperature of the flow-through water baths was also monitored daily throughout the experiment. Oyster mortality was checked daily by tapping on the chamber when the valves of an oyster were open. Tapping the chamber was used to minimize opening the chamber to avoid unnecessary oxygen exchange. If the oyster did not attempt to close its valves after tapping, the chamber was opened, and forceps were used to force the valves to close. The oyster was considered deceased if it was unable to keep its valves closed for longer than 15 s. The build-up of metabolic waste products and bacterial proliferation in static chambers is expected to impact water quality, and therefore ammonia concentration, pH, and the presence of hydrogen sulfide (H_2_S) were measured daily in each chamber following the death of the first oyster and again once all oysters were deceased for that chamber. Water (50 mL) was removed from the chambers and used to determine pH (Sigma pH Test Strips 7.0–14.0), H_2_S (Sigma-Aldrich Hydrogen Sulfide Test Strips: qualitative indicator presence/absence), and ammonia (Fluval Test Aquarium Test Kit). The same volume of water removed for testing was replaced with sterile, anoxic water at the experimental temperature of the chamber.

Two experiments were carried out following the same fully factorial design described above in which temperature and the presence of exogenous bacterial sources were used as factors. Both experiments were identical in setup and are only distinguished by removing deceased oysters in experiment-1 (hereafter: Removal Experiment) and by leaving deceased oysters in the chambers in experiment-2 (hereafter: No-removal Experiment). Following completion of the removal experiment (when all oysters had died), all the equipment was cleaned and re-sterilized. New batches of sterile sand and anoxic sediment were used for the no-removal experiment. To collect oyster tissue for DNA sequencing, the last oyster to die in each chamber (and the last two from one chamber, n = 5 oysters / treatment / experiment) was removed, dissected, and the tissue was placed in ethanol for future sequencing, as described in the following section.

### 16S rRNA analyses of bacterial communities

To describe the bacterial community composition in both experiments, whole oyster tissue was analyzed using 16S rRNA amplicon sequencing as described in Coffin et al. (2021). In brief, for each individual oyster tissue that was preserved in ethanol, tissue was homogenized by hand. A 25 mg subsample of homogenate was used for DNA extraction using the Dneasy Blood and Tissue Kit (Qiagen, Hilden, Germany). The bacterial 16S rRNA gene (V3-V4 region) was amplified with forward and reverse primers (Illumina Inc., San Diego, CA, USA) and Accustart II PCR ToughMix (Quantabio, Beverley, Massachusetts) following the same protocol used in Coffin et al. (2021). DNA sequences were subsequently processed using the 16S Metagenomics workflow in the MiSeq Reporter analysis software (version 2.5.1). Taxonomic classification was performed to the species level using the Illumina-curated version of the Greengenes v13.5 (May 2013); however, the taxonomy results of bacterial assemblages are presented and discussed at the genus level. As the tissue used in this analysis was collected from dead oysters, the bacterial community composition observed may have been influenced by an increase in bacterial proliferation leading to necrosis. Although we aimed to minimize this process by collecting samples within 24 h of death, and immediately preserving tissues in ethanol, this post-mortem analysis limits our ability to directly link bacterial communities observed with the cause of death (Coffin et al. 2021).

### Statistics

The mean number of living oysters was calculated daily for the four different treatments and used to calculate the LT_50_ (time taken for half of the population to die) using the Spearmen-Karber method (R studio) (Hamilton et al. 1977; Babarro and De Zwaan 2008). Survival curves were compared using the non-parametric Kaplan-Meier test to estimate Wilcoxon and log-rank values with a 95% confidence (R Studio) (Kaplan and Meier 1958; Babarro and De Zwaan 2008). When significant differences were observed, pairwise post-hoc analysis with Benjamini-Hochberg correction was used to determine differences between treatments.

For bacterial assemblage data, Bray–Curtis similarity resemblance matrices were created using Plymouth Routines in Multivariate Ecological Research package version 6.1.18 (Clarke and Gorley 2006) with PERMANOVA+ (Anderson et al. 2008). Principal coordinate analysis (PcoA) ordinations were created, using the Bray–Curtis dissimilarity matrices to visualize data by treatment type, e.g., temperature and sediment. PERMANVOAs were used to test between the fixed factors of temperature (20°C or 28°C) and sediment (sterile or anoxic). Similarity percentages were also calculated for each experiment to determine which bacteria contributed most to the dissimilarity between treatments. All statistical analyses were conducted in Rstudio version 4.2.2. Significance for all statistical tests was set at 0.05. The measure of variability reported along with the mean values represents 1 standard error of the mean (mean ± SE) unless otherwise specified.

## Results

### Water quality

Temperature was stable during the experiments for both treatments, 20 ± 0.5 and 28 ± 0.5 °C, respectively. Oxygen levels also remained low and stable in all treatments at 0.01 ± 0.02 mgO_2_L^−1^. pH ranged from 8.0 to 9.0, and from 7.7 to 8.7, in the removal and no-removal experiments, respectively (Table 1). Ammonia concentration increased for both experiments over time, reaching values greater than 6.1mgL^−1^ for most chambers at the end of the experiment. Regarding H_2_S, most of the chambers tested positive for H_2_S only after the onset of mortality (Table 1).

**Table 1:** Summary of experimental conditions for both experiments (removal and no-removal of dead oysters)

Experiment	Temperature	Sediment	LT_50_	Day of First Mortality	H_2_S Presence (After Day)	pH (Average)	Ammonia (mgL^−1^)
Removal	28 °C	Anoxic	9.7 ± 0.5^a^	7	8	8.0– 9.0	Day 7–8:4.6
	28 °C	Sterile	10.9 ± 0.4^ab^	7	8	7.9– 9.0	Day 7–8: 4.6
	20 °C	Anoxic	12.2 ± 0.6^b^	11	12	8.0– 8.9	Day 11–12: > 6.1
	20 °C	Sterile	17.9 ± 0.6^c^	11	12	8.0– 9.0	Day 11–12: > 6.1
No-removal	28 °C	Anoxic	8.0 ± 0.3^a^	6	7	7.7– 8.7	Day 6–8: 4.4
	28 °C	Sterile	9.3 ± 0.4^b^	7	8	7.7– 8.9	Day 6–8: 4.4
	20 °C	Anoxic	9.8 ± 0.4^b^	8	9	7.8– 8.8	Day 9–12: > 6.0
	20 °C	Sterile	13.7 ± 0.4^c^	8	9	7.7– 8.9	Day 9–12: > 6.0

Calculated time taken for 50 percent of the population to die (LT50) for Crassostrea virginica in anoxic (0.01 ± 0.02 mgO2L−1) water under the influence of temperature (20 ± 0.5 or 28 ± 0.5 °C) and exogenous bacterial source (sterile sediment vs. anoxic sediment added at the beginning of the experiment). Letters indicating homogeneous subgroups were calculated using Kaplan–Meier test to estimate Wilcoxon and log-rank values with a 95% confidence within each experiment. H2S, pH, and Ammonia measurements are averages of each experiment. For these water quality parameters, measurements were taken daily after the death of the first oyster in each chamber until all oysters had died (detailed in Sect. 2.3, following Coffin et al. 2021). H2S Presence indicates the day of experiment when H2S was first detected

###  Oyster mortality

**Fig. 1:**
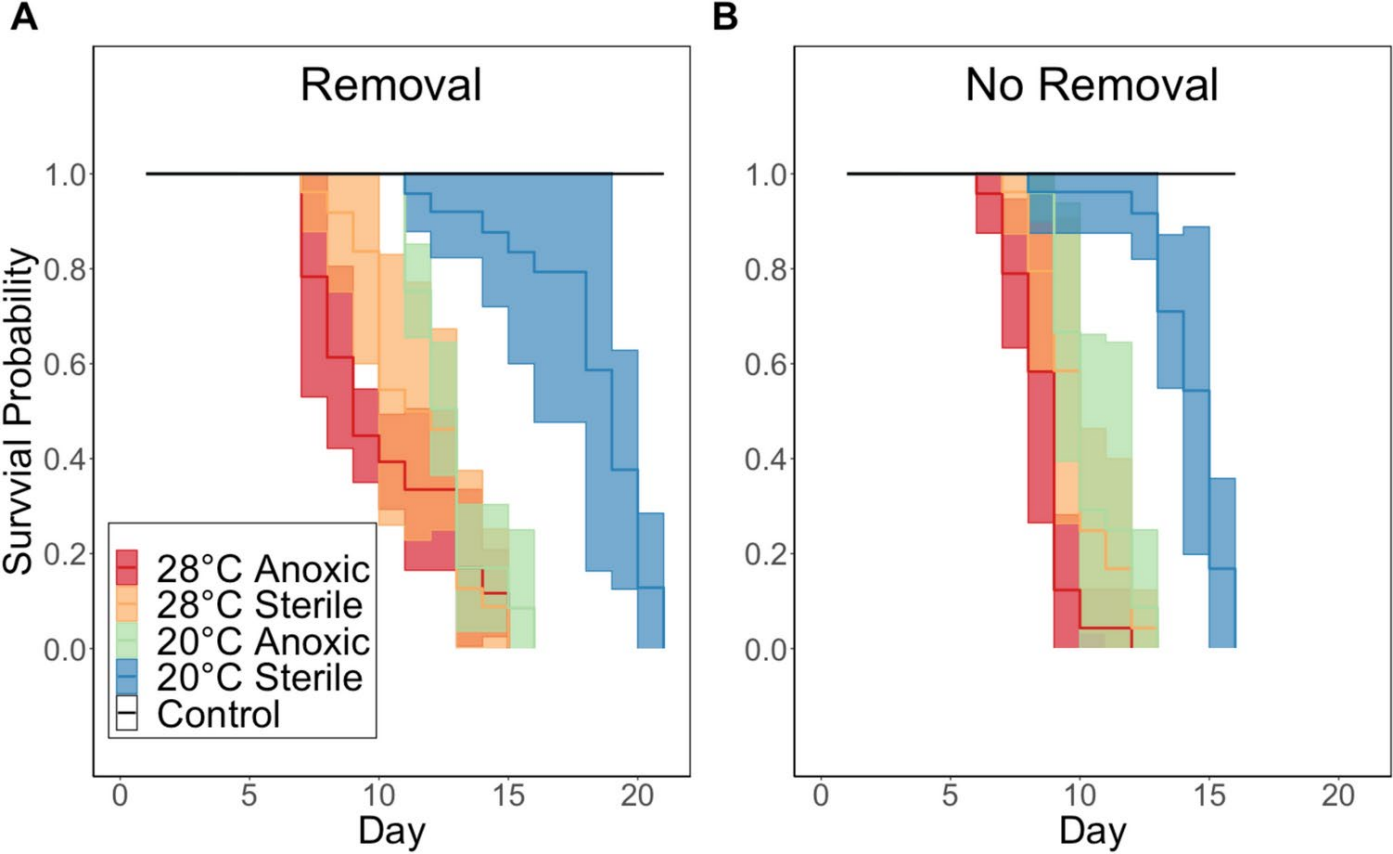
The effect of temperature (20 °C vs 28 °C) and exogenous sediment type (sterile vs anoxic added at the beginning of the experiment) on the survival probability of *Crassostrea virginica* over time (day) in anoxic water with removal of deceased oysters (A, experiment-1) and no-removal of deceased oysters (B, experiment-2). Solid colored lines are the mean survival probability (1 = 100% survival, solid black line), and shaded area is ± 1 standard deviation of each treatment mean

For both experiments, no mortality was observed in the independent normoxic chambers, and similar patterns in mortality rates were recorded between experimental treatments. The first mortalities happened 7 and 6 days after the start of the experiment (onset of anoxia) for the removal and no-removal experiments, respectively (Fig. 1). Higher temperature accelerated mortality rate in both experiments as the 28 °C treatments had initial mortality events occurring earlier than the 20 °C treatments. Similarly, in both experiments, complete mortality occurred later in the 20 °C sterile sediment environment than in any of the other treatments. In the removal experiment, complete mortality for all treatments occurred later than in the no-removal experiment. The LT_50_ values of both experiments cannot be statistically compared given that they were not carried out simultaneously; however, the LT_50_ values (Table 1) are longer when deceased oysters were removed compared to when deceased oysters were not removed.

In the removal experiment, there was an overall significant difference in survival between treatments (*X*^*2*^ (3) = 58.8, p < 0.0001, Fig. 1A). The first oysters died in both 28 °C treatments at the same time (day 7), and from day 7 onwards, the treatment with anoxic sediment caused a slightly higher mortality rate than the sterile sediment; however, both treatments achieved complete mortality the same day (day 15), with no significant differences between LT_50_ values (post-hoc pairwise comparison: p = 0.094, Table 1). Mortality began at the same time in both 20 °C treatments (day 11); thereafter, mortality occurred faster in the treatment with anoxic sediment compared to the sterile sediment, with the former achieving complete mortality shortly after both 28 °C treatments (day 16). Complete mortality in the 20 °C sterile sediment environment occurred several days after the other treatments (day 21), with a significantly longer LT_50_ than all other treatments (post-hoc pairwise comparison: p < 0.01, Table 1, Fig. 1A).

In the no-removal experiment, there was also an overall significant difference in survival between treatments (*X*^*2*^ (3) = 77.3, p < 0.0001, Fig. 1B). The first and last oysters died in both 28 °C environments at almost the same time (first: day 6 and 7, last: day 12 and 13 for anoxic and sterile sediment, respectively); however, the LT_50_ for the sterile sediment treatment was significantly longer (post-hoc pairwise comparison: p < 0.01). For both treatments at 20 °C, the start of mortality closely followed that in the 28 °C treatments (day 8); however, and similarly to the removal experiment, mortality occurred faster in the treatment with anoxic sediment compared to the sterile, with the former having complete mortality on the same day as the 28 °C sterile treatment (day 13). Complete mortality for the 20 °C sterile sediment occurred later than the other treatments (day 16), with a significantly longer LT_50_ time than all other treatments (post-hoc pairwise comparisons: p < 0.01, Table 1, Fig. 1B). Accordingly, the LT_50_ for 28 °C anoxic sediment was the shortest in both experiments while the LT_50_ for 20 °C sterile sediment was the longest (Table 1).

### Bacterial community

**Fig. 2:**
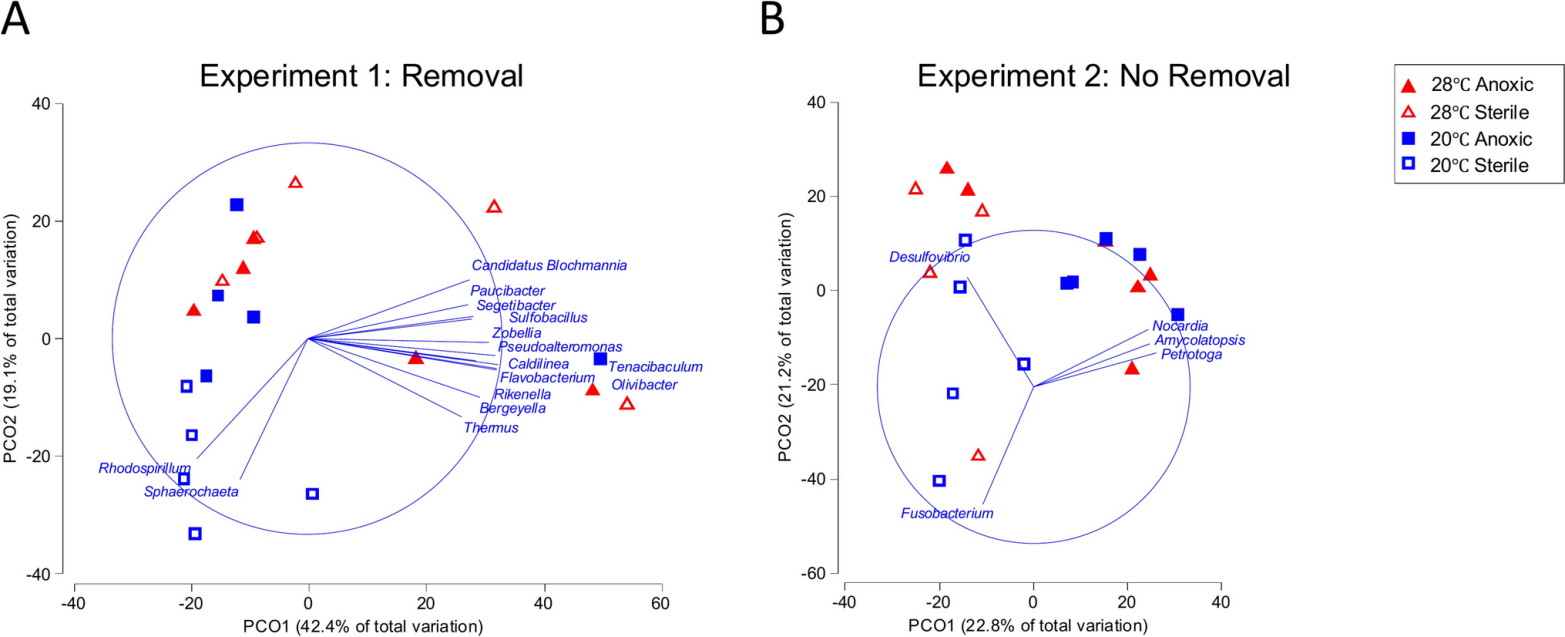
The effect of temperature (20 °C vs 28 °C) and exogenous sediment type (anoxic vs sterile added at the beginning of the experiment)– Principal coordinates analysis using Bray–Curtis similarity indices on the relative abundance of genera of bacteria contained within dead whole oysters, *Crassostrea virginica*, after exposure to sustained anoxia, with removal of deceased oysters (**A**) and no-removal of deceased oysters (**B**). Vector length corresponds to a Pearson’s correlation of r < 0.80. Color and shape denotes temperature (blue squares denoting 20 °C and red triangles denoting 28 °C) and sediment type (open symbols denoting sterile sediment and closed symbols anoxic sediment)

In both the removal and no-removal experiments, there was some discrimination between sterile sediment at 20 °C relative to the other treatments but overall bacterial communities were similar among treatments (Experiment 1: PCO1 = 42.4%, PCO2 = 19.1% of the total variation and, Experiment 2: PCO1 = 22.8%, PCO2 = 21.2% of the total variation, Fig. 2). Relative abundances of bacteria genera were untransformed, meaning that relative abundance differences contributed more to the discrimination between samples than species richness. Indeed, during data exploration, it was discovered that the more severe the transformation, the less discrimination between samples, meaning that the bacterial communities were all similar. Not surprisingly gram-negative, sulfate reducing bacteria were dominant, with differences in the relative abundance of *Desulfovibrio* contributing significantly to the differences between all treatments (Supplemental Table 1).

### Discussion

In this study the effect of anoxia on mortality rates of the oyster *Crassostrea virginica* was explored experimentally using a factorial design, which included the effects of temperature, presence/absence of an exogenous bacterial source, and removing vs. not removing deceased oysters throughout the experiment. High temperature (28 °C vs. 20 °C) significantly increased oyster mortality rates in anoxic conditions. The presence of an exogenous bacterial source (anoxic vs. sterile sediment) increased mortality rates consistently at the low temperature (20 °C), and oyster mortality rates were higher when deceased oysters were not removed throughout the experiment. Generally, bacterial community composition was consistent regardless of anoxic vs. sterile sediment treatment; however, relative abundances of bacterial species differed between treatments. The results of this study provide information about the drivers of oyster mortality during anoxic events, and how temperature and bacteria may interact in low oxygen conditions to impact mortality rates. Considering the increasing frequency of anoxic events in coastal waters, these results may help inform future laboratory experiments, and encourage the monitoring of in situ environmental conditions to better predict bivalve mortality rates.

### Interaction between temperature, bacterial sources, and mortality

Temperature was the most important factor affecting oyster mortality in anoxic conditions; the 28 °C treatments resulted in oyster mortality starting earlier and being completed sooner than in the 20 °C treatments. Bivalve mortality under anoxia is facilitated by anaerobic bacteria that can proliferate under low oxygen conditions, which can also be exacerbated by high water temperatures (Babarro and de Zwaan 2002, 2008; Vezzulli et al. 2013). The effects of bacterial proliferation on oyster mortality can be exacerbated by elevated water temperature may drive immune suppression in bivalves (King et al. 2019). Some of the bacteria causing bivalve mortality under anoxic conditions have been identified in field experiments, and observed to grow best in warm water (e.g., *Vibrio* species > 18 °C) (Garnier et al. 2007; Vezzulli et al. 2013; King et al. 2019). In other oyster species there is also a known positive relationship between mortality risk from herpes-like viruses and herpesviruses (e.g., *Ostreid herpesvirus* 1) and low oxygen availability (Alfaro et al. 2018). Apart from bacterial growth, increasing temperature may also increase the virulence of some of these species (Vezzulli et al. 2013; Guijarro et al. 2015).

It is notable that Rhodospirillum and Sphaerochaeta bacteria were both well correlated with increased survivorship from the 20°C, sterile treatment from the removal experiment. Bacterial information was collected to inform our general hypothesis that there might be specific bacteria associated with mortality. Results from all our experiments demonstrate that membership within the community stays similar but the relative abundances change; however, it is unclear what mechanisms are leading to these changes. Future studies may consider characterizing shifts in bacterial community assemblages over time in anoxic conditions to further elucidate the relationships between bacterial proliferation and mortality.

Oyster mortality rates were consistently higher when deceased oysters were not removed throughout the experiment, particularly at 20 °C with sterile sediment where the LT50 in the removal experiment was almost 4 days earlier. This finding may suggest that even in apparently otherwise non-stressful conditions, the effects of anoxia may be exacerbated by the additional source of bacterial proliferation, and its affiliated decline in water quality (Le Ray et al. 2023). In anoxic conditions, the bacterial consumption and breakdown of organic tissue results in the production of H_2_S, which can alter the carbonate chemistry of bottom waters. The combined effects of low oxygen and the build-up of hydrogen sulfide have been directly linked to mortality in hypoxia experiments (Vaquer-Sunyer and Duarte 2010; Coffin et al. 2021). In this experiment, the H_2_S was not measured quantitatively but in presence/absence; however, Le Ray et al. (2023) observed that H_2_S values exceeded the upper limit for *C. virginica* (279 μM, Riedel et al. 2012) in a hypoxic experiment after the death of all oysters in a mesocosm. As this experiment indicates that not removing dead oysters throughout anoxia accelerates oyster mortality, future experiments may consider examining the causative linkages between necrotic tissue degradation and water quality decline. Further, the high temperature treatment, anoxic sediment treatment only affected oyster mortality in the experiment in which oysters were not removed from the chambers. It could be hypothesized that high temperatures may play a larger role in mortality than the increase in bacterial load under anoxia due to the increase in proliferation and virulence at high temperatures (de Zwaan et al. 2001; Babarro and de Zwaan 2002). The influence of exogenous bacterial sources on oyster mortality, particularly at lower temperatures, indicates that the monitoring of in situ bacterial communities (e.g., composition, activity) may be important in predicting oyster mortality rate during hypoxic events.

When comparing experiments carried out under similar low oxygen concentrations at 20 °C with *C. virginica*, LT_50_ values obtained by Stickle et al. (1989) (20 days), Davis (2021) (~ 25 days), (Davis et al. (2023) (~ 14 days) were similar to the values obtained in this experiment of 17.9 days. However, the LT_50_ values from our experiment at 20 °C with anoxic sediment (12.2 days) are substantially lower than values reported by Stickle et al. (1989) and Davis (2021), and even lower when dead oysters were not removed (9.6 days). When comparing the LT_50_ at 28 °C in this experiment of 10.9 days, with values at 30 °C by Stickle et al. (1989) of 3 days, the differences are remarkable; however, a 2 °C increase in temperature in the upper part of the tolerance range could affect mortality enough to explain the differences in survival times. Thermal tolerance may also be a naturally selected trait, or a hatchery selected trait in *C. virginica*, and as a farmed species with a broad geographic range, the interactive effects of temperature and hypoxia on oyster mortality should be examined on a local scale (Montagnac et al. 2020; Marshall et al. 2021). Further, differences in physiological condition, size, and age can inherently affect the capacity of the oyster to cope with stress, including hypoxia (Garnier et al. 2007; Davis et al. 2023). Accordingly, the absolute LT_50_ values aim to test the effect of the experimental treatments (i.e., hypoxia, bacterial source) rather than providing values that could be directly extrapolated to field conditions across different sources of oysters. Finally, the life cycle stage could play a role in the response as juveniles have been observed to have longer LT_50_ values than larval stages in anoxic conditions (Widdows et al. 1989).

### Limitations and future work

This experiment was conducted in a static system to avoid introducing exogenous factors in water renewal and to preserve the natural bacterial community introduced in the anoxic sediment. As a trade-off, this statis system likely overestimates a realistic duration of long water residence time, no food, and hypoxia. Deterioration of water quality in a static system, due to the accumulation of H_2_S and ammonia may influence bivalve survival (de Zwaan et al. 2001). *C. virginica* is understood to be tolerant to relatively high concentrations of ammonia, with concentrations of > 10mgL^−1^ being required to observe sublethal changes in targeted cellular activity (Keppler 2007). Similarly, in natural systems close to where the anoxic sediment was collected for this study, and where natural oyster populations occur, H_2_S concentration in sediments has been observed to remain consistently high both spatially and temporally (Mallet et al. 2006). pH measurements during this experiment ranged between 7.7 and 9.0, which are similar to the natural oyster habitat in coastal waters of Atlantic Canada (7.1–8.90) (Mayrand and Benhafid 2023). Furthermore, previous studies using a flow-through system rather than a static system have demonstrated similar bivalve mortality rates independently of water quality changes, indicating that water quality plays a minor role in bivalve mortality compared to the effects of bacteria. Additionally, in Coffin et al. (2021), *C. virginica* survival under anoxia was driven by the administration of a general antibiotic in both flow-through and static conditions, suggesting that bacteria contribute more acutely to oyster mortality than water quality. Although treatments including anoxic sediment in this experiment would have introduced an exogenous food source not present in the sterile treatments, it is unlikely that this contributed to oyster survival rates due to the behavioural response of oysters to close their valves, and thus stop feeding, in response to anoxia (Coffin et al. 2021). Similarly, treatments including anoxic sediment may have also contained heavy metals that tend to accumulate in industrialized coastal marine habitats (Kibria et al. 2021). As heavy metal exposure is understood to interactively cause physiological stress to marine bivalves under anoxic conditions, future studies interested in hypoxia and mortality should also consider monitoring heavy metal contamination in anoxic sediments (Lannig et al. 2008; Ivanina et al. 2011).

This study suggests that multiple stressors may contribute to oyster mortality during anoxia, and that the design of laboratory experiments is important for extrapolating to realized mortality rates in field conditions; however, there is a lack of baseline information about water quality and bacterial communities during anoxic events in oyster growing areas. Field observations of water quality before, during, and after anoxic events are needed to corroborate laboratory findings and inform future experiments. For example, water quality parameters (e.g., temperature, dissolved oxygen, ammonia, pH, H_2_S) should be regularly measured in oyster habitats or aquaculture farms where anoxic events often occur during the summer. Further, bacterial assemblage analyses be conducted on sediment samples and oyster tissues before, during, and after anoxic events. In situ sampling of bacterial communities would avoid any possible disturbances to the communities during the collection and transport of the anoxic sediment to the laboratory. Differences and similarities between sediment and oyster tissues could be compared both temporally, but also by oyster proximity to the sediment (e.g., natural reefs, bottom culture, and floating gear). With a more accurate understanding of changes in water quality during anoxic events, more realistic laboratory experiments can be conducted to observe oyster physiology and mortality and further understand the mechanisms of mortality during anoxic events. This work would improve our capacity to predict the effects of these events on wild and farmed bivalve populations.

### Conclusion

The factorial design of this study highlights the importance of exploring interacting environmental stressors associated with anoxic conditions as drivers of bivalve mortality under anoxia. As expected, temperature had a strong effect on oyster mortality under anoxia, with the greatest survival occurring at lower temperatures. At higher temperatures, the presence of an exogenous bacterial source from anoxic sediment did not have a large effect on oyster mortality as temperature seemed to have a greater effect, dominating the dynamics of the mortality event. Conversely, at a lower temperature having anoxic sediment in the experimental chambers increased oyster mortality rates. Further, oyster mortality occurred more rapidly when dead oysters were not removed throughout the experiment, suggesting that dead oysters could provide additional substrate for bacterial proliferation. Results from this study highlight the need for future experiments to observe in situ anoxic events to further our understanding of the mechanisms of mortality in oysters in anoxic conditions. This research is important for predicting bivalve mortality rates, in both natural and farmed populations during concurrent warm and anoxic events, which are predicted to become increasingly common in many coastal waters under climate change scenarios.

## Supplementary Information

Below is the link to the electronic supplementary material.

Supplementary file1 (PDF 149 kb)

## Data Availability

Datasets generated in this study are available from the corresponding author by request.
