# Supplementary material for: The combined effects of temperature and exogenous bacterial sources on mortality in the Eastern oyster (Crassostrea virginica) under anoxia
Source: Mar Biol. 2025 Mar 17;172(4):57. doi: 10.1007/s00227-025-04617-4 (PMC11913911; doi:10.1007/s00227-025-04617-4)
Supplement: Supplementary file 1 — Supplementary file1 (PDF 149 kb) [file 227_2025_4617_MOESM1_ESM.pdf]

Supplemental Table 1 for:

The combined effects of temperature and exogenous bacterial sources on mortality in the Eastern oyster (*Crassostrea virginica*) under anoxia

Laura Steeves[1†], Keryn Winterburn[2†], Michael R.S. Coffin[3], Jose M. F. Babarro[4], Thomas Guyondet[3], Luc A. Comeau[3], Ramón Filgueira[5]

[1]Department of Marine and Coastal Sciences, Rutgers University, Port Norris, New Jersey, USA

[2]Biology Department, Dalhousie University, Halifax, Nova Scotia, Canada

[3]Fisheries and Oceans Canada, Gulf Fisheries Centre, Moncton, New Brunswick, Canada

[4]Instituto de Investigaciones Marinas, IIM-CSIC, Vigo, Spain

[5]Marine Affairs Program, Dalhousie University, Halifax, Nova Scotia, Canada

†These authors contributed equally to this work and share first authorship

Supplemental Table 1. Results of SIMPER analyses describing the top genera contributing to the dissimilarity between oysters exposed to different temperatures (20 or 28°C) and sediment types (sterile or anoxic) for the removal and no removal experiments, respectively). The cutoff of genera of bacteria was arbitrarily determined at contributing ~5% of the contrasting taxa and was 6 genera for all treatment combinations.

**Experiment**		**Average relative abundance**		**Average dissimilarity**	**Contrasting taxa contribution (%)**
**Removal**	Genera	20°C	28°C		
**Sterile**	*Sphaerochaeta*	18.28	0.98	8.65	14.01
	Bacteriovorax	15.31	0.81	7.25	11.75
	Flavobacterium	2.64	13.74	6.37	10.31

	Rikenlla	1.41	10.50	5.14	8.33
	Alkaliphilus	11.42	5.85	3.96	6.42
	Desulfovibrio	9.77	8.40	3.65	5.91
**Anoxic**	*Flavobacterium*	8.58	12.67	7.29	14.22
	Rikenella	5.74	9.07	5.53	10.79
	Desulfovibrio	3.05	8.63	3.55	6.93
	Alkaliphilus	6.66	8.55	3.39	6.62
	Unclassified	21.33	15.33	3.34	6.52
	Bacteroides	7.58	7.86	2.71	5.29
**No Removal**					
**Sterile**	*Fusobacterium*	13.67	4.66	6.89	12.67
	Desulfovibrio	13.05	17.14	4.98	9.17
	Unclassified	17.09	19.23	3.90	7.17
	Alkaliphilus	13.75	6.15	3.85	7.09
	Flavobacterium	0.99	8.13	3.8	6.99
	Treponema	6.46	1.92	2.50	4.61
**Anoxic**	*Desulfovibrio*	10.68	26.38	10.54	21.08
	Unclassified	32.06	18.08	7.71	15.43
	Clostridium	9.42	17.51	7.70	15.41
	Rhodococcus	8.33	2.22	3.24	6.48
	Bacterioides	3.75	7.46	2.83	5.66

Arcobacter	1.56	4.14	1.85	3.69
